# Epigenetic RELN Dysfunction in Schizophrenia and Related Neuropsychiatric Disorders

**DOI:** 10.3389/fncel.2016.00089

**Published:** 2016-04-05

**Authors:** Alessandro Guidotti, Dennis R. Grayson, Hector J. Caruncho

**Affiliations:** ^1^Department of Psychiatry, The Psychiatric Institute, College of Medicine, University of Illinois at ChicagoChicago, IL, USA; ^2^College of Pharmacy and Nutrition, University of SaskatchewanSaskatoon, SK, Canada

**Keywords:** RELN, synaptic plasticity, promoter methylation, schizophrenia, bipolar disorder, Dab1

## Abstract

REELIN (RELN) is a large (420 kDa) glycoprotein that in adulthood is mostly synthesized in GABAergic neurons of corticolimbic structures. Upon secretion in the extracellular matrix (ECM), RELN binds to VLDL, APOE2, and α3β2 Integrin receptors located on dendritic shafts and spines of postsynaptic pyramidal neurons. Reduced levels of RELN expression in the adult brain induce cognitive impairment and dendritic spine density deficits. RELN supplementation recovers these deficits suggesting a trophic action for RELN in synaptic plasticity. We and others have shown that altered RELN expression in schizophrenia (SZ) and bipolar (BP) disorder patients is difficult to reconcile with classical Mendelian genetic disorders and it is instead plausible to associate these disorders with altered epigenetic homeostasis. Support for the contribution of altered epigenetic mechanisms in the down-regulation of RELN expression in corticolimbic structures of psychotic patients includes the concomitant increase of DNA-methyltransferases and the increased levels of the methyl donor S-adenosylmethionine (SAM). It is hypothesized that these conditions lead to RELN promoter hypermethylation and a reduction in RELN protein amounts in psychotic patients. The decreased synthesis and release of RELN from GABAergic corticolimbic neurons could serve as a model to elucidate the epigenetic pathophysiological mechanisms acting at pyramidal neuron dendrites that regulate synaptic plasticity and cognition in psychotic and non-psychotic subjects.

## Introduction

REELIN (RELN) is an extracellular matrix (ECM) glycoprotein that controls neuronal cell migration and the lamination of the corticolimbic structures during embryonic development (D’Arcangelo et al., 1995). RELN also plays a role in controlling dendritic spines, and synapse structure and function in adulthood (Costa et al., 2001). Research in the last 20 years, has suggested that abnormal brain RELN expression is a feature that associates with major neuropsychiatric disorders including schizophrenia (SZ), bipolar (BP) disorder (Impagnatiello et al., 1998; Fatemi et al., 2000; Guidotti et al., 2000), autism (Fatemi, 2002), depression (Lussier et al., 2009, 2011, 2013), and Alzheimer’s disease (Herz and Chen, 2006). While the role of RELN in dendritic spine structure, synapse plasticity, and cognitive function in adulthood has been extensively studied, considerably less research has focused on the mechanisms whereby RELN expression is altered in neuropsychiatric conditions. Here, we review evidence for a role of the epigenetic control of the expression of RELN in the regulation of neuronal plasticity and behavior in SZ and BP disorder patients compared with controls devoid of major psychiatric disorders.

## RELN in the Adult Mammalian Brain

### Neuronal Location

**Figure 1:**
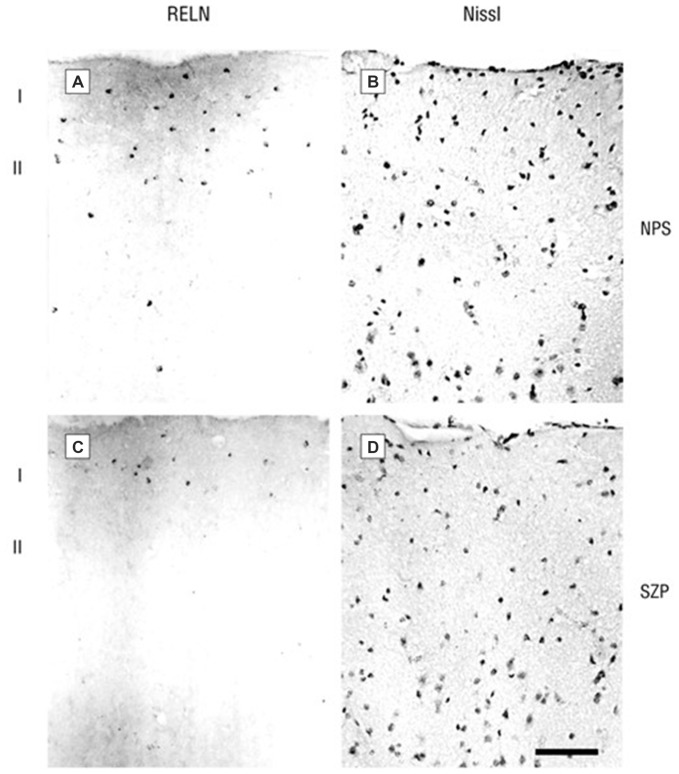
**Photomicrographs of 20 μm sections of prefrontal cortex (PFC) of a non-psychiatric subject (NPS) and of a schizophrenia patient (SZP) immunolabeled for RELN (A,C, left side) or Nissl-stained (B,D, right side).** RELN positive neurons are mostly localized in layer 1. Note that the NPS has a higher density of RELN-positive cells and also a stronger extracellular diffuse RELN immunostaining halo. Reprinted with permission from Guidotti et al. (2000).

In the cortex and hippocampus of adult rodents and primates, RELN is predominantly synthesized and secreted by GABAergic interneurons (Alcántara et al., 1998; Impagnatiello et al., 1998; Pesold et al., 1998, 1999; Guidotti et al., 2000; Rodriguez et al., 2000; Kadriu et al., 2012). Immunohistochemistry coupled to *in situ* hybridization studies distinguishes at least two sets of GABAergic interneurons based on their ability to synthesize and secrete RELN. The first synthesizes and secretes RELN onto apical and basal dendrites of pyramidal neurons and includes GABAergic horizontal, double bouquet, multipolar and Martinotti neurons in layers 1 and 2 of the mammalian cortices (Figure 1). The second set of GABAergic neurons, which do not usually express RELN, include chandelier and basket interneurons that innervate the axon initial segment or somata of pyramidal neurons, respectively (Pesold et al., 1998, 1999). In contrast, in cerebellum, RELN is predominately synthesized by glutamatergic granule neurons, and is secreted by their parallel fiber axon terminals into the ECM surrounding the dendrites of GABAergic Purkinje cells (Pesold et al., 1998). Studies in primary cultures of rat cerebellar granule cells suggest that RELN is secreted in the extracellular medium in a manner that is blocked by the constitutive secretory pathway inhibitor brefeldin. Moreover, secretion of RELN is independent of neuronal activity (Lacor et al., 2000). These findings suggest the possibility that secretory pathway activators might be useful in facilitating RELN secretion when RELN expression is compromised.

### Extracellular Location

Once released in the extracellular space, RELN binds to VLDL, APOE and α3β1 integrin receptors activating the signal transduction system in the effector cells including apical and basilar dendrites of pyramidal neurons in the neocortex or Purkinje cells in the cerebellum (D’Arcangelo et al., 1999; Hiesberger et al., 1999; Dong et al., 2003; Strasser et al., 2004).

**Figure 2:**
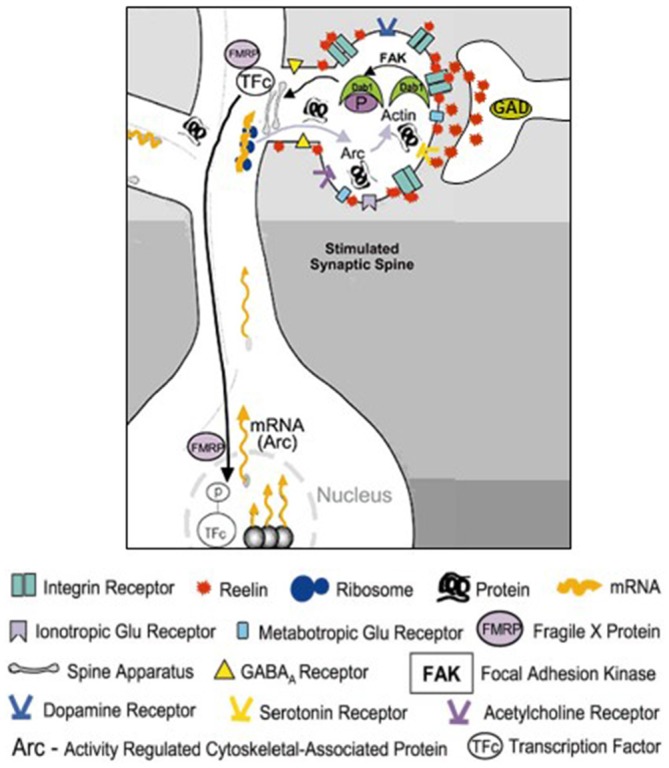
**Schematic representation of the RELN signaling pathways in dendritic spines.** RELN released in the extracellular matrix (ECM) from the terminal of cortical GABAergic neuron binds to dendritic shafts and spines and modulates the transcriptional function of activated dendritic synapses. Modified from Costa et al. (2001). RELN binding to Integrin receptors located on dendritic spines mediates activation of focal adhesion kinase (FAK) that directly or indirectly, via activation of Fyn kinase, phosphorylates DAB1 adaptor protein resident in dendrites. Phosphorylated dendritic DAB1 may recruit or activate ribosomal structures and induce the synthesis of ARC (Activity regulated cytoskeletal-associated protein) or cytoskeletal proteins like β-actin.

Using electron microscopic techniques, Costa et al. (2001) demonstrated the presence of RELN-like immunoreactivity decorating the dendritic shafts and spines of distal apical dendrites of pyramidal neurons in the frontal cortex. This area, as well as the hippocampal fissure, is characterized by strong diffuse RELN–immunoreactivity (Pesold et al., 1998). The colocalization of RELN with the α3 subunit of the integrin receptor at post-synaptic densities of adult rat and primate brains suggests that a RELN signaling mechanism involving integrin and VLDL and APOE2 receptors may be operative in modulating the strength of synaptic function (Rodriguez et al., 2000; Dong et al., 2003; Niu et al., 2004). It has been shown that RELN interacting with VLDL, APOE2, or integrin receptors results in activation of the Src-tyrosine kinase family Fyn-kinase, leading to tyrosine phosphorylation and recruitment of the cytoplasmic adaptor protein DAB1 (Figure 2; Jossin et al., 2003; Bock et al., 2004; Kuo et al., 2005). Studies suggest that DAB1 phosphorylation is a crucial step in the activation of RELN signal transduction pathways (Rice et al., 1998; Trommsdorff et al., 1999; Niu et al., 2004; Howell and Pillai, 2015). DAB1 is frequently expressed in proximity of synapses located on dendritic spines or shafts of cortical pyramidal neurons (Rodriguez et al., 2000). Hence, phosphorylated DAB1 may regulate cytoskeletal protein synthesis at dendrites by activating the translation of dendritic resident mRNAs (see Figure 2, from Costa et al., 2001).

## RELN Regulates Spine Density and Excitatory Synaptic Function

Animal models in which RELN expression is genetically reduced provide important information on the impact of RELN on synaptic plasticity and cognition. *Reeler* mice display altered LTP and deficits in active avoidance tasks (Goldowitz and Koch, 1986; Marrone et al., 2006). Young adult heterozygous *reeler* mice (HRM) which exhibit a 50% reduction in RELN content have significantly reduced dendritic spine densities and also show a deficit in LTP (Tueting et al., 1999, 2006; Liu et al., 2001; Levenson et al., 2008; Niu et al., 2008; Iafrati et al., 2014). Adult HRM also have a defective molecular composition of the synaptic structure (Ventruti et al., 2011), as well as deficits in excitatory postsynaptic responses to glutamate receptor agonists and reduced LTP (Levenson et al., 2006). Addition of recombinant RELN to hippocampal slices or direct injection of RELN into the cerebral ventricles enhances hippocampal LTP (Beffert et al., 2006; Pujadas et al., 2010; Rogers et al., 2011).

HRM also display deficits in cognitive function (Krueger et al., 2006; Stranahan et al., 2011), executive function (Brigman et al., 2006), fear conditioning learning (Ammassari-Teule et al., 2009), anxiety and motor impulsivity (Ognibene et al., 2007). Importantly, RELN supplementation recovers sensory motor gating, synaptic plasticity, and associative learning deficits in HRM (Rogers et al., 2013). In addition to the HRM data described above, Lussier et al. (2013) reported that hippocampal RELN deficiency elicited by chronic stress (repeated corticosterone treatment) can impair adult hippocampal neurogenesis and lead to the development of a depression-like phenotype. Co-treatment with antidepressant drugs prevents both RELN deficit and the development of the depression-like phenotype (Fenton et al., 2015).

Addition of recombinant RELN to cortical synaptosomes *in vitro* induces the expression of activity-regulated cytoskeleton-associated protein (Arc; Dong et al., 2003), and augments the density and clustering of proteins in postsynaptic membranes (i.e., neurotransmitter receptors), which provides further evidence of a functional role for RELN in regulating the synaptic strength of glutamatergic inputs onto dendritic spines (Caruncho et al., 2004).

## RELN in the Brains of Schizophrenia (SZ) and Bipolar (BP) Disorder Patients

A number of molecular, anatomical (dendritic spine density), behavioral, and cognitive deficits associated with reduced RELN expression (mRNA and protein) are observed in subjects with SZ and BP disorder. In different post-mortem brain cohorts, we and others have demonstrated that RELN expression is reduced by approximately 50% in the prefrontal cortex (PFC), temporal cortex, hippocampus, and caudate nucleus of patients with SZ and BP disorder (Impagnatiello et al., 1998; Fatemi et al., 2000; Guidotti et al., 2000). In successive studies we found that the expression of RELN was paralleled by decreases in the levels of GAD67 but not DAB1 or GAD65. Slices from the same samples immunostained for RELN and counterstained for Nissl or NeuN to recognize neurons showed that RELN-positive neurons were significantly decreased by 30–50% in patients with SZ or BP disorder with psychosis but not in those with unipolar depression when compared to non-psychiatric subject (NPSs; Figure 1). Differences were absent for GAD65, and NeuN expression implying that RELN and GAD67 down-regulation is unrelated to neuronal damage (Guidotti et al., 2000). The RELN and GAD67 downregulation is also unrelated to postmortem interval, dose, duration, or presence of antipsychotic medication. Similar to HRM, RELN deficiency in the neocortex of SZ and BP disorder patients is associated with a decrease in GAD67, reduced prepulse inhibition to startle, and loss of dendritic spines, all features associated with SZ pathology (Tueting et al., 1999; Glantz and Lewis, 2001; Liu et al., 2001; Grayson and Guidotti, 2013).

Like the RELN deficiency in the cerebellar cortex of HRM, the RELN deficiency in cerebellar cortex of SZ and BP disorder patients is associated with a 20% decrease of GABAergic Purkinje neurons (Hadj-Sahraoui et al., 1996; Maloku et al., 2010). Collectively these data suggest that RELN plays a central role in inducing and maintaining the structure and function of GABAergic and glutamatergic neurons and their reciprocal interactions (Grayson and Guidotti, 2013).

Since SZ and BP disorder have a neurodevelopmental origin (Folsom and Fatemi, 2013) and RELN is a major player in brain development and maturation (D’Arcangelo et al., 1995), an important question raised by these studies is whether the altered epigenetic (promoter hypermethylation) regulation of RELN in brains of SZ and BP patients is initiated early in embryonic or perinatal life or develops later in life as the consequence of the GABAergic neuropathology related to the development of SZ morbidity. To address this question, the extent of methylation of the RELN promoter was measured in offspring born from mice stressed during pregnancy. These offspring, at adulthood, display SZ-like behavioral endophenotypes (increased locomotor activity, PPI, social recognition deficits), and a decrease of RELN, GAD67, and BDNF expression associated with an increase in methylation at their respective promoters. We also found that the amount of Methyl CpG Binding Protein 2 (MECP2) binding to the *RELN* promoter at birth was higher than that observed in the adult (Matrisciano et al., 2013). These data suggest that RELN promoter hypermethylation is likely initiated early in life, including during embryonic life, and is then maintained throughout adulthood.

## Is an Altered Epigenetic Regulation of Gene Expression the Molecular Mechanism Mediating RELN Expression Down-Regulation in SZ and BP Disorder?

Mutations in the RELN gene are associated with a form of autosomal recessive lissencephaly with abnormal axonal connectivity, and cerebellar hypoplasia (Hong et al., 2000). Human subjects with RELN gene mutations exhibit marked ventricular dilation, mental retardation, and epilepsy and a marked decrease in muscle tone that appears of neurogenic origin (Hourihane et al., 1993). Heterozygous RELN mutations have been shown to cause autosomal-dominant lateral temporal epilepsy (Dazzo et al., 2015).

A highly conserved single nucleotide polymorphism (SNP) has been identified in the vicinity of the regulatory region of the RELN gene (Shifman et al., 2008; Wedenoja et al., 2010). This polymorphism is associated with an increased risk of psychotic symptoms. Although these studies highlight the importance of RELN gene variants as risk factors in the etiopathogenesis of psychiatric disorders, it is important to note that variants in the RELN gene are rare and cannot explain the high frequency of RELN expression downregulation observed in the general population of SZ, BP disorder and autism spectrum disorder patients (Zhang et al., 2002; Lintas and Persico, 2010; Grayson and Guidotti, 2013; Wang et al., 2014; Zhubi et al., 2014).

The epidemiological and clinical evidence that SZ and BP disorders do not follow the rules expected for a Mendelian-genetic disorder led to the proposal that environmental insults may influence RELN gene expression by altering epigenetic regulatory mechanisms and led to the hypothesis that epigenetic factors are operative in mediating changes in the expression of RELN and other SZ candidate genes in psychotic patients (Costa et al., 2003). To better understand the rules governing the epigenetic regulation of *RELN*, we cloned the human gene and experimentally examined its regulation in both neuroprogenitor NT2 cells (Chen et al., 2002; Mitchell et al., 2005) and mouse cortical neurons *in vitro* (Dong et al., 2003; Noh et al., 2005). Data from these studies support the concept that the *RELN* promoter is regulated epigenetically through changes in DNA methylation. Furthermore, we have reported that the down-regulation of RELN expression in GABAergic neurons of SZ and BP patients is associated with an overexpression of DNA methyltransferase 1 (DNMT1) and DNA methyltransferase 3a (DNMT3a) in neocortical and striatal GABAergic neurons (Veldic et al., 2004, 2007; Ruzicka et al., 2007). DNMTs are a family of enzymes that catalyze the transfer of a methyl group from the methyl donor S-adenosylmethionine (SAM) to the 5′ carbon of cytosine of many gene promoters (Grayson and Guidotti, 2013). Increased promoter methylation generally leads to decreased gene expression. Interestingly, the inhibitory action of DNMTs on RELN expression also likely occur through the formation of chromatin repressor complexes which include, in addition of DNMTs, also the methyl CpG binding domain proteins, SIN3A, and histone deacetylases (see Grayson and Guidotti, 2013 for review).

The hypothesis that an epigenetic pathology of the *Reln* promoter is operative in the transcriptional down-regulation of the corresponding gene in SZ or BP disorder patients is supported by the evidence that there is an increased level of SAM in the PFC of these patients (Guidotti et al., 2007), and that hypermethylation of the *RELN* promoter (Abdolmaleky et al., 2005; Grayson et al., 2005, 2006; Lintas and Persico, 2010) is associated with the down-regulation of the corresponding protein in the PFC of psychotic patients (Guidotti et al., 2000), although negative findings for RELN promoter hypermethylation have also been reported (Mill et al., 2008).

In other studies a decreased histone methylation at GABAergic gene promoters (Huang et al., 2007), and an increased histone deacetylase -1 expression and down regulation of GABAergic gene expression in PFC and hippocampus of SZ patients have been reported (Benes et al., 2007; Sharma et al., 2008). A summary of many of the methylation studies of *RELN* in neuropsychiatric patients, SZ-like epigenetic mouse models, and neuronal culture systems are summarized in Table 1. These data are consistent with the epigenetic GABAergic theory of major psychosis (Costa et al., 2003; Grayson and Guidotti, 2013) and suggest that* RELN* promoter methylation should be further studied to establish its temporal and casual association with the etiopathogenesis of SZ and BP disorder.

**Table 1:** **Summary showing studies of RELN methylation relevant to neurobiology^a^**.

Reference	Location^b^	Species	Tissue	Design	Method	Result
Abdolmaleky et al. (2005)	Promoter: below −700 bp	Human	Frontal Lobe	RELN methylation in SZ vs. Con were compared	Bisulfite seq, Methylation specific PCR	*RELN* promoter is hypermethylated in SZ
Aberg et al. (2014)	First Intron	Human	Whole Blood	RELN methylation in SZ vs. Con were compared	Methyl Binding Domain- profiling	*RELN* is hypermethylated in SZ
Blaze et al. (2013)	Promoter	Rats	Medial Prefrontal Cortex	Comparison of methylation status at the *Reln* promoter as a function of post-natal maltreatment or nurturing care	Methylation specific PCR	*Reln* methylation varies by condition, age and sex
Chen et al. (2002)	Promoter: below −527 bp	Human	NT2 Cells	*RELN* methylation in differentiated vs. control neuroprogenitor cells *in vitro* were examined	Bisulfite seq	*RELN* hypermethylation in NT2 cells that is demethylated on differentiation
Dong et al. (2007)	Promoter: −520 to −198 bp	Mouse	Frontal Cortex	Methionine (MET) induced hypermethylation of the *RELN* promoter was examined and the effects of VPA and MS-275 on this methylation *in vivo* were evaluated	Methylation specific PCR	MET induces *RELN* methylation, while VPA and MS-275 reverse this methylation
Dong et al. (2016)	Promoter: −220 to +70 bp	Mouse	Frontal Cortex	PRS mice were examined for changes in *Reln* promoter methylation or hydroymethylation at PND 75 following either vehicle (VEH), clozapine (CLZ) or haloperidol (HAL)	MeDIP, hMeDIP	At PND 75, PRS strongly induces RELN promoter hypermethylation and, to a lesser extent hydroxyl methylation, of the *Reln* promoter. CLZ, but not HAL, attenuates the PRS-induced hypermethylation
Grayson et al. (2005)	Promoter: below −527 bp	Human	BA 9 and 10	*RELN* methylation in SZ vs. Con were compared	Bisulfite seq	*RELN* is hypermethylated in SZ
Kobow et al. (2009)	Promoter: below −500 to +100 bp	Human	Hippocampus	Human temporal lobe epilepsy (TLE) biopsy specimens vs. autopsied control tissue were compared	Bisulfite seq	*RELN* promoter hypermethylation was observed in TLE biopsies
Kundakovic et al. (2009)	Promoter: below −250 bp	Human	NT2 Cells	The effect of the HDAC inhibitor, MS-275, on RELN promoter methylation was determined	MeDIP followed by qPCR	MS-275 induces *RELN* promoter demethylation
Levenson et al. (2006)	Promoter: between −1000 and −500 bp	Mouse	Hippocampal Slice Preparation	The response of neurons in slices to the effects of Protein Kinase C (PKC) activation by phorbol esters and or by inhibitors of DNA methylation	Methylation specific PCR	Reln promoter methylation is decreased by inhibitors of DNA methylation and activators of PKC
Lintas and Persico (2010)	Promoter: below −413 bp	Human	BA 41 and 42	*RELN* promoter methylation was examined in pre- and post-pubertal post-mortem brain from non-psychiatric subjects	Bisulfite seq	Post-pubertal *RELN* promoter is hypermethylated compared with pre-pubertal *RELN*
Matrisciano et al. (2011)	Promoter: −423 to −252 bp	Mouse	Frontal Cortex	PRS mice were examined for methylation vs. non-stressed mice and the effect of LY379268 on this methylation	MeDIP	PRS mice showed *Reln* promoter methylation which was reduced by LY379268
Matrisciano et al. (2013)	Promoter: −432 to −252 bp	Mouse	Frontal Cortex	PRS mice were analyzed for changes in Reln promoter methylation and hydroxymethylation vs. Con	MeDIP and hMeDIP	PRS mice showed elevated Reln promoter methylation and hydroxymethylation at PND 60
Mill et al. (2008)	Promoter	Human	Frontal Cortex	*RELN* methylation in SZ vs. BD vs. Con were compared	Pyrosequencing	No change between groups
Mitchell et al. (2005)	Promoter: below −500 bp	Human	NT2 Cells	Determined the effects of HDAC and DNMT inhibitors on *RELN* promoter methylation	Bisulfite seq	TSA, VPA and AZA induces *RELN* hypomethylation
Noh et al. (2005)	Promoter: −340 to +140 bp	Mouse	Cortical Neurons	MET was used to manipulate *RELN* promoter methylation *in vitro*	Bisulfite seq	MET induced *RELN* promoter hypermethylation
Palacios-Garcia et al. (2015)	Promoter: −786 to −625 bp	Rats	Whole Cortex Cultured Neurons	PRS rats were analyzed for changes in *Reln* promoter methylation *in vivo* and *in vitro*	Methylation sensitive restriction enzyme PCR	*Reln* promoter methylation is increased in newborn PRS rats and in cultured neurons *in vitro*
Qin et al. (2011)	Promoter	Rat	Hippocampus	The effects of maternal deprivation on *Reln* promoter methylation were examined	Methylation specific PCR	Maternal deprivation facilitated increased *Reln* promoter methylation
Sui and Li (2010)	Promoter: −700 to −400 bp	Rat	Hippocampus	Promoter methylation was analyzed in rats with perinatal hypothyroidism at PND 1 through 60	Methylation specific PCR	Hypothyroid rats show elevated *Reln* promoter methylation at PND 1, 5 and 15 relative to Con
Sui et al. (2012)	Promoter	Rat	Medial Prefrontal Cortex	Promoter methylation was analyzed following the induction of LTP as compared with Con	Methylation specific PCR	High frequency stimulations induce DNA demethylation at the *Reln* promoter vs. Con
Tremolizzo et al. (2002)	Promoter: −340 to +160 bp	Mouse	Frontal Cortex	The effects of VPA treatment on the MET-induced hypermethylation of the *RELN* promoter were evaluated	Bisulfite seq	Methionine induces *RELN* methylation, while VPA reverses this effect
Zhubi et al. (2014)	Promoter: −220 to +70 bp	Human	Cerebellum	Reln promoter methylation and hydroxymethylation were analyzed in autism spectrum disorder (ASD) vs. typically developed subjects (Con)	MeDIP and hMeDIP	While *RELN* promoter methylation levels are unchanged between ASD and Con, 5hmC content at the promoter is increased

*^a^The above studies do not include at least one report (Tochigi et al., 2008) that showed no detectable RELN promoter methylation in either SZ or control subjects by pyrosequencing. In addition, there are numerous studies showing that elevated RELN promoter methylation is associated with poor prognosis in various types of cancers. We apologize for any relevant studies that were inadvertently omitted from this list. ^b^For precise locations of the RELN methylation see the associated reference. The locations provided are approximate and if no coordinates are indicated, then the information was not in the original report. For example, Promoter (without additional information) indicates that the authors specified the RELN promoter without giving additional coordinates. ASD, Autism spectrum disorder; Bisulfite seq, Bisulfite sequencing; CLZ, Clozapine; Con, Control; HAL, Haloperidol; hMeDIP, Hydroxymethyl DNA immunoprecipitation; LTP, Long-term potentiation; MeDIP, Methyl DNA immunoprecipitation; NT2 cells, Ntera2 cells; PCR, Polymerase chain reaction; PKC, Protein kinase C; PND, Post-natal day; PRS, Pre-natal restraint stress; TLE, Temporal lobe epilepsy; VEH, Vehicle; VPA, Valproic acid*.

## RELN, Spine Density Down Regulation and Cognitive Performance Deficits Induced by L-Methionine Treatment

Support for the hypothesis that an increase of DNA methylation contributes to the down-regulation of RELN and other GABAergic or glutamatergic genes in psychotic patients is sustained by clinical studies conducted in the early 1970s (for review see Wyatt et al., 1971; Cohen et al., 1974; Grayson et al., 2009). In these studies L-methionine (MET, the precursor of SAM), administered in large doses (10–20 g/day) for 3–4 weeks to SZ patients was reported to exacerbate psychotic symptomatology (Cohen et al., 1974; Grayson et al., 2009). Patients were administered large doses of L-methionine either with or without a monoamine oxidase inhibitor in an attempt to reduce the levels of putative bioactive psychedelic compounds. Interestingly, many of the treated patients responded with a worsening of their symptoms (Cohen et al., 1974).

**Figure 3:**
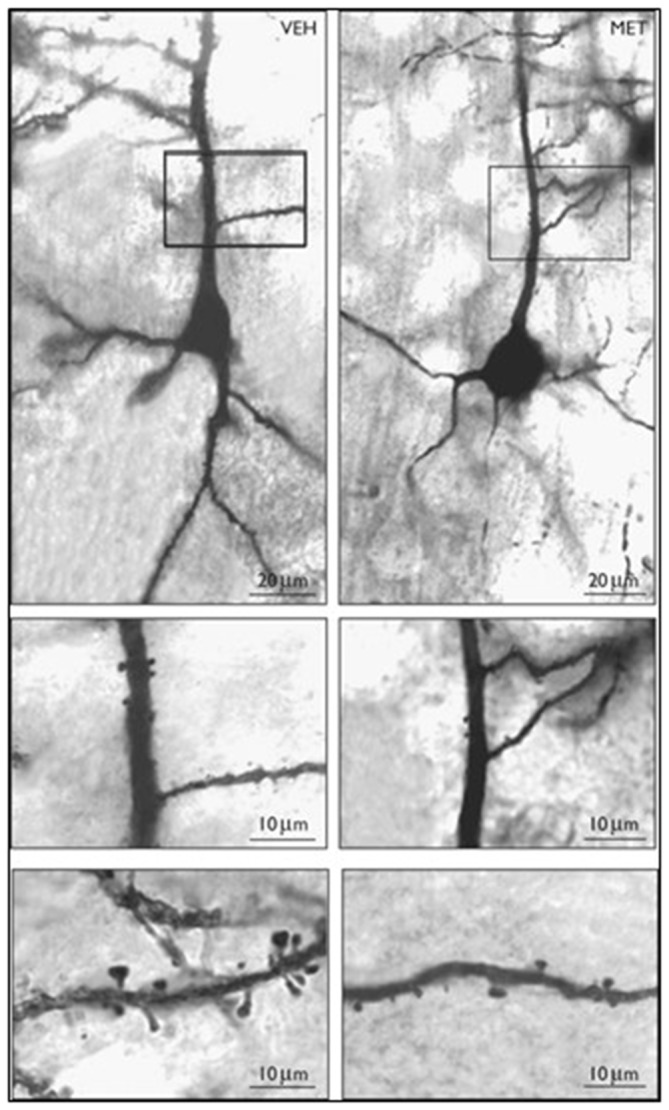
**Photomicrographs of Golgi stained layer III pyramidal neurons in vehicle (VEH) and methionine (MET) treated mouse frontal cortex.** Top panel: 10× objective; Middle panel: 20×; Bottom panel: 100× Vehicle or L-methionine (5.2 mmol/kg/twice a day) was administered for 14 days. Reprinted with permission from Tueting et al. (2010).

In both mouse FC and neuronal cultures, the administration of large doses of L-methionine increases SAM levels and facilitates the hypermethylation of GABAergic gene promoters, including *Reln*, and *GAD67* and the reduced expression of these genes (Tremolizzo et al., 2002, 2005; Mitchell et al., 2005; Noh et al., 2005; Chen et al., 2007). Similar to the HRM, spine density is also decreased in the dendrites of mice treated with L-methionine (Figure 3, Tueting et al., 2010). Furthermore, L-methionine treated mice display SZ-like behavioral abnormalities (Tremolizzo et al., 2005). Collectively, these data suggest that the reduction of dendritic spines observed in brain of L-methionine-treated mice are likely due to MET-induced altered epigenetic mechanisms that lead to decreased expression of RELN (Tremolizzo et al., 2005; Tueting et al., 2010).

## RELN Promoter Methylation in Neurons is a Dynamic Process that can be Targeted by Environmental Factors and Drugs

The dogma that in post-mitotic neurons DNA methylation patterns are established during development and remain stable thereafter (Razin and Shemer, 1995) has been challenged by convincing evidence that in post-mitotic neurons, methylation patterns of specific cytosine/guanine (CpG) dinucleotide-rich promoters, change rapidly. Thus, in neurons, promoter methylation provides a series of targets on which the environment, drugs, and/or toxins can modify transcription and affect neuronal phenotype profiles without altering the genotype (Szyf, 2009). To verify this hypothesis, we treated (Tremolizzo et al., 2002, 2005; Tueting et al., 2010) mice protractedly with L-methionine (as described above) and measured the ratio of 5 methyl cytosine (5mC) to unmethylated cytosine (C) of the murine RELN CpG-enriched promoter region from −340 to +160 bp (Tremolizzo et al., 2005) or the murine GAD_67_ CpG-enriched promoter region from −760 to −311 bp (Satta et al., 2008) by measuring the fraction of promoters immunoprecipitated by specific anti-5mC or anti-MeCP2 antibodies with competitive RT-PCR and internal standards (Dong et al., 2005). We found that (Dong et al., 2005; Tremolizzo et al., 2005) methionine induces an increase of brain RELN and GAD_67_ promoter methylation (Dong et al., 2005), and downregulation of RELN and GAD_67_ mRNA and cognate protein expression associated with decreased spine density (Figure 3), and SZ-like behavioral modifications (Tremolizzo et al., 2002, 2005; Tueting et al., 2010). The effects of methionine on the RELN promoter, RELN protein level, dendritic spine density, and SZ-like behavioral modifications are reversed by the administration of Valproic acid (VPA) and other HDAC inhibitors (Dong et al., 2005; Tremolizzo et al., 2005). These findings, together with data obtained in the HRM, suggest the working hypothesis that the down-regulation of spine density and SZ-like behavioral modifications in L-methionine treated mice may be, in part, due to decreased expression of RELN.

## Concluding Remarks

SZ and BP are neurodevelopmental disorders with genetic risk load and behavioral and neurochemical SZ-like phenotypes triggered by exposure to prenatal or perinatal environmental insults: stress, toxins, infection, trauma. In mice exposed prenatally to restraint stress, we found increased DNMT levels that are associated with RELN promoter hypermethylation, RELN expression downregulation, SZ-like epigenetic behavioral modifications, and decreased dendritic spine density in adultood (Tremolizzo et al., 2005; Tueting et al., 2010; Dong et al., 2016). L-Methionine supplementation in rats induces epigenetic variations including RELN promoter hypermethylation in offspring (Weaver et al., 2005). Further, there is an epigenomic reprogramming of RELN and glucocorticoid receptors in hippocampal pyramidal neurons after methionine administration (Weaver et al., 2006). Our studies in cultured mouse cortical neurons (Noh et al., 2005) and human neuronal progenitors (Kundakovic et al., 2007, 2009) not only show that the hypermethylation of promoters induced by L-methionine is blocked by siRNA-mediated DNMT-KO or by reduction of DNMT activity with small molecule antagonists but also that this blockade induces the overexpression of RELN, GAD_67_, or BDNF proteins (Kundakovic et al., 2007, 2009).

Collectively, these data challenge the classic concept that 5-methylcytosine patterns in DNA remain stable in post-mitotic neurons and strongly suggest that by increasing brain SAM content, L-methionine facilitates the promoter methylation mediated by DNMT1 or DNMT3a in the central nervous system (Grayson and Guidotti, 2013). Unlike the DNA sequence of a cell, which is stable and strongly conserved, epigenetic processes that impact DNA methylation and chromatin architecture are highly dynamic. That is, they can be tissue-specific, developmentally-regulated, and modified by a wide range of drugs and other environmental factors (Szyf, 2009; Ptak and Petronis, 2010; Grayson and Guidotti, 2013; Dong et al., 2016).

Studies using the L-methionine mouse model or offspring of restraint stressed mothers may be aimed at determining whether antipsychotics capable of reducing RELN promoter methylation (e.g., clozapine), enhance spine density, and relieve SZ-like epigenetic behaviors (Tremolizzo et al., 2005; Dong et al., 2016). These models should provide useful preclinical tools for screening small molecules for their capacity to reverse SZ candidate gene promoter methylation and the associated neuronal and behavioral deficits.

## Author Contributions

All authors contributed equally to the ideas and editing of the manuscript.

## Funding

AG is supported by the following NIH grants AA022538, R01 MH093348, R01 MH101043, DRG is supported by AA022538. HJC is supported by a SHRF Establishment Grant, and a NSERC Discovery grant.

## Conflict of Interest Statement

The authors declare that the research was conducted in the absence of any commercial or financial relationships that could be construed as a potential conflict of interest.
